# Targeting of Androgen Receptor Expression by Andro-miRs as Novel Adjunctive Therapeutics in Prostate Cancer

**DOI:** 10.4236/jct.2013.44A006

**Published:** 2013-04

**Authors:** Jey Sabith Ebron, Crystal M. Weyman, Girish C. Shukla

**Affiliations:** 1 Center for Gene Regulation in Health and Disease, Cleveland State University, Cleveland, USA; 2 Department of Biological, Environmental Sciences, Cleveland State University, Cleveland, USA

**Keywords:** Androgen Receptor, microRNA, 3’ Untranslated Region, Prostate Cancer, Castration-Resistant Prostate Cancer (CRPC), Andro-miR

## Abstract

Prostate cancer begins as an androgen-responsive disease. However, subsequent accumulation of multiple sequential genetic and epigenetic alterations transforms the disease into an aggressive, castration-resistant prostate cancer (CRPC). The monoallelic Androgen Receptor (AR) is associated with the onset, growth and development of Prostate cancer. The AR is a ligand-dependent transcription factor, and the targeting of androgen- and AR-signaling axis remains the primary therapeutic option for Prostate cancer (PCa) treatment. A durable and functional disruption of AR signaling pathways combining both traditional and novel therapeutics is likely to provide better treatment options for CRPC. Recent work has indicated that expression of AR is modulated at the posttranscriptional level by regulatory miRNAs. Due to a relatively long 3’ untranslated region (UTR) of AR mRNA, the posttranscription expression is likely to be regulated by hundreds of miRNAs in normal as well as in disease state. The main objective of the article is to offer a thought-provoking concept of “andro-miRs” and their potential application in AR gene expression targeting. This new paradigm for targeting constitutively active AR and its tumor specific splicing isoforms using andro-miRs may pave the way for a novel adjunctive therapy and improved treatment of CRPC.

## 1. Introduction

Prostate Cancer (PCa) is the most commonly diagnosed malignancy among the male population in North America and continues to be the second leading cause of cancer-related deaths [1]. Recent statistics estimate that PCa alone account for 28% of all cancers in male population. PCa cells depend on androgens, mainly testosterone and dihydrotestosterone (DHT), for their growth and survival. Androgens are responsible for development of male characteristics during embryogenesis and later the maintenance of male sexual behavior and reproduction [2]. Dysregulation in the action of androgens has been associated with the development of PCa; and hence, the standard therapeutic intervention for PCa has been Androgen Receptor (AR) directed therapeutics, achieved through androgen-deprivation or chemical castration. Though this method is initially effective, recurrent castration-resistant prostate cancer (CRPC) arise, for which there is no reliable means of treatment.

Acquired resistance to anti-androgen therapy remains a major obstacle in the treatment of advanced stage metastatic cancer. Although the exact molecular mechanisms responsible for the progression of the disease from androgen-dependent state to androgen-refractory state is unclear, CRPC tumors display revived capability of androgen signaling through AR, a ligand dependent transcription factor belonging to nuclear receptor super-family [3,4]. Testosterone is the most commonly present androgen in prostate cells and is bound in its inactive form with carrier proteins such as albumin or Sex hormone-binding globulin [5]. In prostate cells, testosterone is converted into its more potent form DHT by the action of 5-α reductase [6]. Both testosterone and DHT exert their action by binding to AR protein in cytoplasm.

The AR protein structure is composed of three major functional domains: N-terminal transactivation domain (TAD), central DNA-binding domain (DBD) and C-terminal ligand-binding domain (LBD) [7,8]. Molecular binding of DHT to the LBD stimulates the transcriptional activity of the AR, which then regulates the expression of numerous androgen-responsive genes with myriad of cellular functions. Specifically, binding of ligand induces a conformational change leading to localization of AR homodimer in the nucleus, where it binds to androgen-responsive elements (ARE) in the promoter of target genes through its DNA-binding domain that results in activation of a complex transcriptional program. In CRPC, constitutive AR modulated signaling pathways are crucial for their association with metastatic PCa [9].

In this review, we briefly remind the reader of potential mechanisms mediated through AR; though we mainly intend to focus on the implication of miRNAs mediated regulations of PCa, with AR-centric approach. We discuss the regulation of AR activity through andro-miRs and their potential application as therapeutic agents in treatment of CRPC patients.

## 2. Androgen Receptor—In the Center of Prostate Carcinogenesis

Discovery of a crucial role for AR in PCa dates back to the 1940s when Huggins and Hodges demonstrated the effectiveness of surgical castration in men with PCa [10]. Since then, hormonal blockade has been the mainstay of PCa therapy. Currently, androgen deprivation is achieved through a combination of drugs such as Gonadotro-pin-releasing hormone (GnRH) agonists and/or AR antagonists. Altered expression of AR is identified with almost all primary and metastatic PCa [11]. Downstream influence of AR signaling is also used in the diagnosis of PCa by detecting the increased serum level of Prostate Specific Antigen (PSA). Changes in AR signaling are used to monitor the success of treatments such as androgen-ablation and also to monitor the progression of the disease to CRPC [12]. Studies have shown that AR is expressed in the vast majority of CRPC tumors, is transcriptionally active, and is required for tumor cell growth [13]. Thus, AR is indisputably pivotal in the progression of the disease from the androgen-dependent state to castration resistant state in which the tumors dependent upon AR signaling through complex cellular mechanisms that circumvent traditional androgen deprivation therapy (ADT) and AR antagonists [14,15].

### 2.1. Androgen Receptor Gene Amplification

Majorities of PCa are dependent on AR signaling—even as they progress into castration-resistant state; AR continues to remain the favorite target for cancer therapy. Overexpression of AR at both the mRNA and protein levels has been reported in CRPC tumors [16,17]. AR gene amplification occurs in approximately 25% – 30% of CRPC tumors which had undergone traditional androgen-deprivation therapy while the untreated PCa saw no such consequences [18]. AR amplification therefore represents a molecular mechanism that has potential to cause hormone therapy failure and definite development of CRPC. Androgen-dependent and independent PCa cells respond to alterations in the AR protein level. Upregulation of the levels of AR in these cells promotes proliferation, while inhibition of AR signaling induces apoptosis [19]. Further, increased levels of AR sensitized CRPC cells to residual levels of androgens [20]. However, there are reports showing AR gene amplification independent of overexpression of AR protein; suggesting carcinogenic regulation involving epigenetic mechanisms and/or miRNA [21]. These findings confirm the significance of optimal AR signaling in the survival and proliferation of CRPC tumors; hence targeting of the AR signaling pathway remained an excellent strategy for treating advanced stage PCa.

### 2.2. Androgen Receptor Gene Mutations

Accumulation of a significant number of AR mutations is another molecular mechanism exploited by PCa cells to acquire androgen-independence and become castration-resistant. AR gene is one of the most abundant mutated of the nuclear steroid receptor family with 1,029 mutations have been reported in the AR gene mutations database (http://androgendb.mcgill.ca). Over 800 of these mutations appear to promote androgen -independence [22]. The incidences of AR mutations are much higher in advanced stages cancer (10% – 20%) compared to that of initial stages (0% – 4%). In addition, multiple AR mutations were detected in the same patient. This finding distinctly applies that clonal selection favors the survival of cells that display multiple AR gene mutations [22].

The first AR gene mutation (T877A) was identified in hormone-dependent LNCaP PCa cells derived from lymph-node metastasis [23]. Interestingly, most mutations, including T877A, accumulate in the ligand-binding domain of the AR protein. These mutations results in decreased ligand specificity, constitutive AR expression and AR promiscuity. A few of the commonly found AR mutations leading to promiscuity include V715M, H874Y and T877S resulting in stimulation of AR by progesterone, T877S and H874Y both induced by estradiol and V715M; and L701H+T877A resulting in stimulation of AR by adrenal androgens and hydrocortisone [24]. Loss-of-function mutations have also been identified in AR; resulting in a weaker response to androgens and failure to trans-activation of target genes in the presence of DHT. Surprisingly, most of the mutations which lead to androgen-independence are found in Exon 1; a large exon, coding for more than half of the protein. It is interesting to note that, very few mutations are identified in the splicing and untranslated regions of the AR gene [22].

### 2.3. Alternatively Spliced Variants of Androgen Receptor

Several AR splice variants have been identified and altered splicing has emerged as another significant molecular mechanism potentially contributing to the recurrence of PCa. AR splice variants AR3, AR4, AR4b, AR5 and AR8 lacks the LBD and have been found to be over expressed in CRPC tumors [25]. The absence of LBD has been implicated in constitutively active AR expression and its nuclear localization characteristic that is independent of ligand binding. This molecular mechanism appears to compensate for the absence of androgens in tumors undergoing hormonal ablation therapy, and thereby promoting androgen-independent tumor growth [26,27]. These splice variants appears to be involved in drug resistance against AR inhibitors like Enzalutamide (MDV3100) [28].

## 3. Androgen Receptor Co-Activators and Suppressors

AR protein is known to interact with a plethora of diverse proteins including other transcription factors as well as AR coregulators with distinct coactivating or corepressing properties. Interaction of AR with coactivator proteins is important for assembly of the transcriptional complexes to modulate transcriptional activities of its target genes. A recent update of AR-database registers over 300 proteins as AR coregulators. As of February 2013, the list of proteins that have been identified as AR-coregulators in the Nuclear Receptor Signaling Atlas (NURSA) contains 283 entries. These coregulators are involved in a multitude of functions. The first AR co-activator to be identified was SRC-1 [29]. The steroid receptor coactivator family containing SRC-1, SRC-2 and SRC-3 contains histone acetyl transferase activity. These co-activators interact with AR in a ligand-dependent manner to initiate transactivation of AR-regulated genes [30,31]. These coactivators along with other components like CBP and P/CAF, acetylate AR in the DNA binding domain hinge region [32]. The binding of these coactivators are important for AR transactivation, failing which results in a 10 fold increase in the binding of the suppressor protein NCoR (nuclear receptor corepressor). NCoR recruits histone deacytylases leading to chromatin packing and further reduction in transcriptional activity of the receptor [33].

Although the list of proteins functioning as coregulators for AR is vast, AR may regulate the expression of most of the coregulators, since modulation of expression of the cofactors are dependent on an AR feedback mechanism to regulate the coregulator complex formation [34]. Alteration in the balance of coregulatory proteins and their binding to AR thus provides a growth advantage to PCa cells and may also play a role in the recurrence of disease.

## 4. Development of Androgen-Independence—Potential Roles of miRNAs

In the last few years, there have been numerous reports supporting the impact of microRNAs (miRNAs) in the progression of androgen-sensitive PCa to CRPC. Only recently, however a couple of reports have shown that the AR is a direct target of miRNAs [35,36]. Regulation of AR expression at the posttranscriptional level by miRNA is an area of increasing curiosity in the context of AR regulation in PCa. miRNAs, along with RNA-based molecules including ribozymes [37,38], short hairpin RNA (shRNA) [39], small interfering RNA (siRNA) [40,41] and antisense oligonucleotides [42,43] present promising alternative to repress AR protein expression by targeting AR mRNA.

miRNAs are short (~20 – 24 nucleotides) noncoding RNAs that regulate gene expression by facilitating cleavage of its target mRNA in plants; and mostly by translational repression in animals. In animals, miRNAs target mRNAs by imperfect complimentary base-pairing to the 3’ untranslated region (3’ UTR) to downregulate target’s protein synthesis in actively translating ribosomes [44]. Currently, miRBase has a compilation of 2042 mature human miRNAs which are predicted to target > 45,000 sites that account for nearly 60% of human genes [45,46]. miRNAs play an important roles in cellular processes such as development, differentiation, proliferation, apoptosis and metabolism. Further, aberration in miRNAs activities has been implicated in human disease pathogenesis [47]. Pioneering studies on *C. elegans* and *D. melanogaster* in the 1990s have shed light on the role of miRNAs in carcinogenesis [48]. In the last few years, aberrant and differential expression of miRNAs has been identified in different stages of tumor pathogenesis including metastasis. Additionally, differential miRNA expression appears to play a crucial role in the prognosis of various cancers including PCa [49]. Studies miRNAs show that about 50% of the miRNA genes are located at sites of frequent amplification, deletion, and CpG island methylation—suggesting that dysregulated expression of these miRNAs is an important factor in tumorigenesis in nearly all types of cancers [50].

Our knowledge of miRNA mediated regulation of PCa still presents an unclear picture owing to the heterogeneity of the disease and the complexity of cellular signaling presumably involved in the conversion of androgen-sensitive to CRPC. Expression profiling of miRNAs in androgen-dependent versus androgen-independent cell lines reveal that miRNA expression is significantly increased in metastatic invasive cell lines such as the PC3, DU145, and MDA PCA 2B indicating the importance of miRNAs in the progression of the disease to androgen-independence [51]. Of the numerous miRNAs shown to be expressed in PCa cells, many have been associated with AR-mediated signaling. miR-221 and miR-222 are the two most commonly overexpressed oncogenic miRNAs (oncomirs) in various cancers including CRPC. These miRNAs target p27 and in turn promote constitutive cell-cycle regulation [52,53]. Studies have shown that overexpression of these miRNAs in the androgen-de-pendent LNCaP cell line can lead to androgen-independence and reversal of the effect was observed by silencing these miRNAs [54]. miR-146 appears to be downregulated in androgen-independent cell lines and overexpression of it leads to decrease cell proliferation and survival, classifying this miRNA as a tumor suppressor [55]. Another important candidate miRNA involved in CRPC is miR-125b. Though studies have shown differential regulation of miR-125b during progression to CRPC; it is known to negatively regulate the expression of HER-2/neu, a central tumor suppressor in breast cancer. CRPC tumors exhibit upregulation of HER-2/neu levels suggesting the oncogenic potential of miR-125b [56–58]. In summation, these studies suggest that miRNAs may have a role as oncogenesis promoting as well as tumor-suppressors molecules in different stages of the development of CRPC.

Several other miRNAs have been reported in PCa including miR-126^*^, miR-330, miR-148a and miR-449. Each of these affects the expression of growth regulatory genes in PCa [59–61]. Upregulation of miR-141 has been detected in PCa cell lines and it has been shown to increase AR transcriptional activity by repressing small heterodimer partner Shp, a corepressor of AR [62]. Conversely, miR-let-7c has been identified as a negative regulator of AR expression by targeting its transcription by c-myc [63]. Similarly, miR-331-3p has been identified to negatively regulate AR signaling pathway through downregulation of the ERBB-2 tyrosine kinase receptor [64]. Collectively, these findings corroborate the importance of miRNAs in the expression of AR as well as in the development of androgen-sensitive PCa and its transition to fatal CRPC.

Greater than 50% of miRNA genes are coded in the introns of protein coding genes. Some studies have shown that there appears to be a coordinated expression of the host genes and the miRNAs that are coded in the introns, because most intronic miRNAs must be transcribed from the host gene’s RNA polymerase II type promoters [65]. Nevertheless, post transcriptional processing of miRNAs, especially those coded in the cluster format, must have evolved to find a way of differential “intra-clustery” expression. Interestingly, a large number of intronic miRNA expressions appear to be uncoordinated with the expression of its host gene, especially in abnormal intrinsic cellular situations. Expression of the C13orf25 gene which contains a polycistronic miRNAs cluster in intron 3 code for six miRNAs (miR-17-5p, miR-19b, miR-20a, miR-92, miR-18a and miR-19a), does not appear to correlate to expression of these miRNAs [51]. This situation appears to support the notion that the differential expression of aberrantly expressed miRNAs may be due to differential posttranscriptional processing of miRNAs in tumors of different origin. Within this cluster mature miR 17-5p, miR-19b, miR-20a and miR-92 appear to be highly expressed in androgen-sensitive LNCaP and MDA PCa 2b cells. Their expression, however, is noticeably downregulated in androgen-refractory cells such as PC3 and DU145. Interestingly, miR-18a and miR-19a expression, although part of the same cluster, is downregulated in both, LNCaP and MDA PCa 2b cells and almost no expression was observed in PC3 and DU145 cells. Similarly, uncoordinated expression between the host-gene AMPO and its intronic miRNAs (miR-23b, miR-27b and miR-24-1) was observed. Differential regulation of members of polycistronic cluster miRNAs may be playing an important role in acquiring androgen-independence in PCa. In this context, some validated target mRNAs of miR 17 – 92 cluster members in C13orf25 include tumor suppressors such as p21, Bim, PTEN, Rb2/p130, Rb12 as well as oncogenes such as E2F1 and AIB [66–70]. Most of these genes also play important roles in AR mediated Prostate carcinogenesis. Therefore, miRNA mediated regulation, or lack-there-of, has immense potential to promote cellular proliferation mediated via AR signaling leading to androgen-independence and CRPC.

## 5. Targeting Androgen Receptor Expression by miRNAs in Prostate Cancer—A Potential Adjunctive Therapeutics

Numerous miRNAs express differentially in PCa cells and large number of these miRNAs have potential to target androgen-centric signaling pathways in addition to targeting of AR expression directly. An experimental undertaking to identify all the miRNAs, which have potential to directly interact with AR 3’ UTR is clearly lacking. A validation of all the miRNAs targets found within the 3’ UTR of AR and fine-tuned influence of these andro-miRs in AR expression is of paramount importance to realistically understand the progression of disease to metastatic stages. It is interesting to note that a modest 3–4 fold increase in expression of AR expression is sufficient for tumors to develop androgen-independence [71], and downregulation of this slight increase in AR protein expression via miRNAs expression is likely to bring much needed therapeutic respite [72,73].

As noted earlier, due to a relatively large 3’ UTR, the AR is likely to be targeted by a plethora of miRNAs. It is logical that all the predicted andro-miRs may not express in prostate cells or in tumors. Nevertheless, it is expected that experimental introduction of miRNAs into PCa cell or tumors is likely to downregulate AR expression. An elegant recent study by Östling *et al.*, using a *tour de force* approach analysis of miRNAs targeting AR expression, revealed 71 potential andro-miRNAs that down-regulated endogenous AR protein levels in multiple AR positive PCa cell culture models [35]. Among the 71 miRNAs the study indentified, 19 miRNAs were shown to have significant downregulatory effect on AR expression. Furthermore, the study demonstrated direct interactions of 13 andro-miRNAs with target binding sites found within the 3’ UTR of AR, using target-validation Luciferase reporter assays. However, it is not clear if other miRNAs are directly targeting the AR or the effect is indirectly manifested by off-target activities of miRNAs. Therefore, target-validation of all of these newly identified andro-miRs is extremely crucial to fully appreciate the impact of miRNAs in PCa. Another study demonstrated that miR-488^*^ can directly bind to the 3’ UTR of the AR to downregulate AR expression both at the mRNA and protein levels resulting in decreased cell proliferation and increased apoptosis in LNCap and C4-3B cells [36]. Our recent work shows that miR-644 binds to target sequences in the 3’ UTR of the AR and downregulates Luciferase expression in target-validation experiments in addition to downregulation of endogenous AR expression in PCa cells (personal communication).

A comprehensive study to biochemically catalog miRNAs that have the potential to bind to the AR 3’ UTR would help the PCa and AR research community to start thinking towards potential application of miRNA based adjunctive therapy of PCa. However, off-target effect of miRNAs should also be considered cautiously in order to establish a comprehensive picture of miRNA mediated PCa therapy.

**Figure 1:**
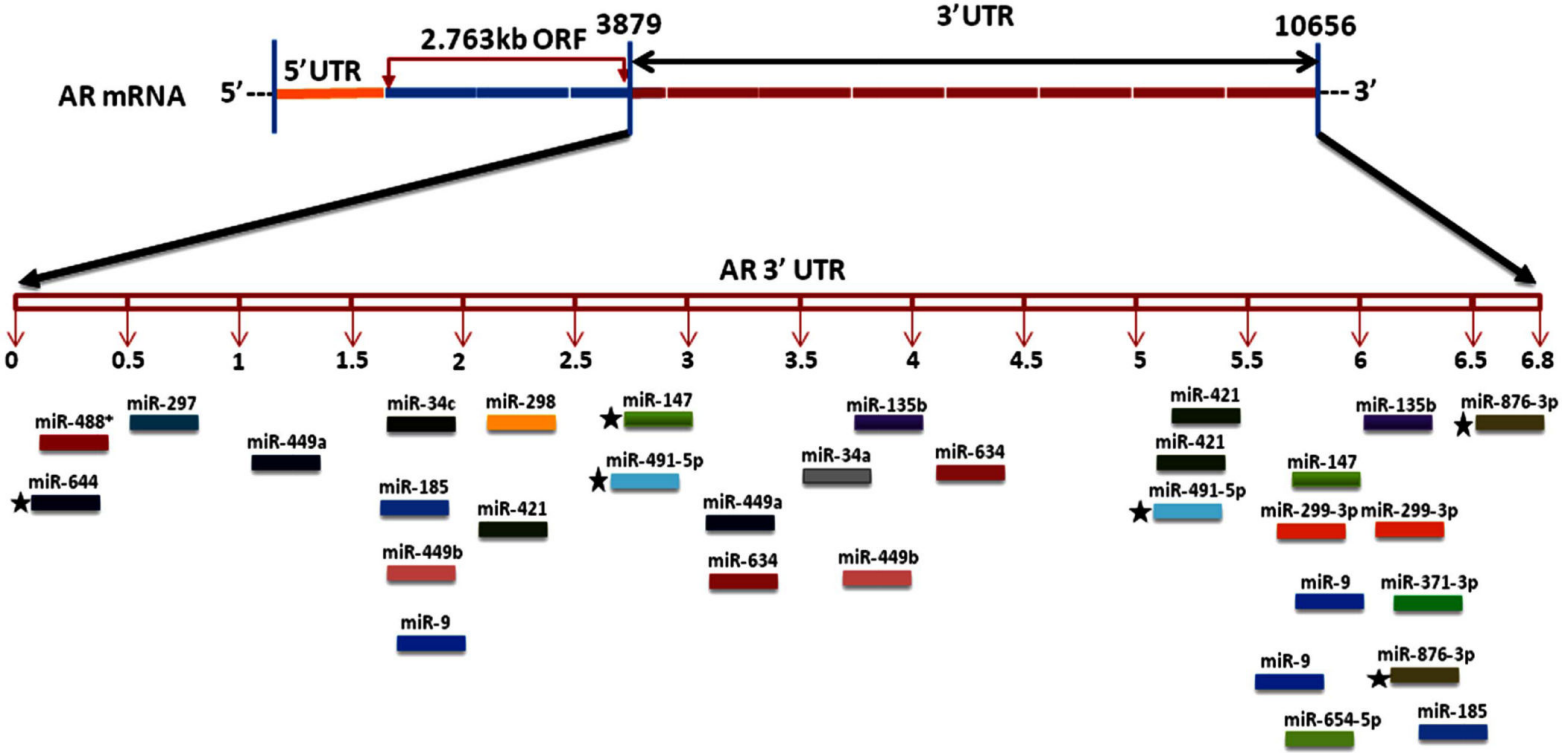
This figure depicts the relative location of andro-miR target sites on AR 3’ UTR schematically. AR mRNA is shown in the top panel with its 5’ UTR, open reading frame and 6.8 kb 3’ UTR

We propose that miRNAs which have been validated to interact directly with the AR 3’ UTR by base-pairing and downregulate AR mRNA and protein levels, can be conveniently termed as andro-miRs, albeit in PCa context only. A list of target-validated andro-miRs is shown in Table 1 along with their binding coordinates on AR mRNA. Figure 1 shows schematic relative position of potential andro-miRs (from Table 1) binding sites in the 3’ UTR of AR.

AR mRNA species of length 8.5 kb and 11 kb with variable 6.6 to 6.9 kb to 3’ UTR lengths have been identified [35,57,74], however only until recently human genome browser reported a 436 nucleotide long 3’UTR [RefSeq: NM_000044] [74–76]. The existence of long AR 3’ UTR proposes a definite possibility of complex regulation of AR expression through an increased number of miRNAs and possibly other auxiliary factors including 3’ UTR regulatory proteins as well as alternative polyadenylation signals. AR mRNAs isoforms with variable 3’ UTR lengths complicates the situation, where in the experimental determination of specific miRNA interactions with different AR isoforms would be a daunting task. Nonetheless, it would be worthwhile to establish the role of specific miRNAs in the fine-tuning of AR expression in prostate carcinogenesis. Additionally, andro-miRs have potential to be utilized as bio-markers of prostate carcinogenesis as well as their potential application in PCa therapy.

## 6. Conclusion

Decades of research have presented us with complex scenarios for the development of CRPC; however, the role of androgen and AR in this progression is irrefutable. The heterogeneity and inherent complex molecular circuitry of disease continues to rely on androgen and its cognate receptor, leading to drug-resistance and failed-treatments. Because of these intrinsic biological properties, the current practice of targeting the AR protein activity does not appear to be completely effective. Development of curative therapies for the treatment of CRPC is urgently needed; especially when resistance to AR-antagonist Enzalutamide (MDV3100) poses a grim prognosis. Discovery of andro-miRs has opened up a new avenue, which must be further explored to establish the efficacy of miR-based adjunctive therapeutics in CRPC treatment. This approach would require the development of an efficient systemic delivery method for andro-miRs to directly target the expression of AR mRNA isoforms, in addition to conventional therapeutics which are designed to target androgen and the AR protein. Last but not least, the 3’ UTR of the AR remains an uncharted territory needing further exploration to understand the regulatory role of this region: the important cis-acting RNA motifs and structural elements, as well as the alternative polyadenylation events responsible for the activation of multiple isoforms. Understanding the RNA-processing events and their potential role in AR function would comprehensively enhance our knowledge of this very fascinating, yet under studied molecule.

## Figures and Tables

**Table 1:** Andro-miRs that are known to downregulate AR expression in PCa cells by direct interaction to AR 3’ UTR: The lists includes the miRNAs that have been validated for their AR:miR RNA-RNA binding experiment using heterologous Luciferase reporter constructs in Sikand *et al.* 2011 and Östling P *et al.* 2011.

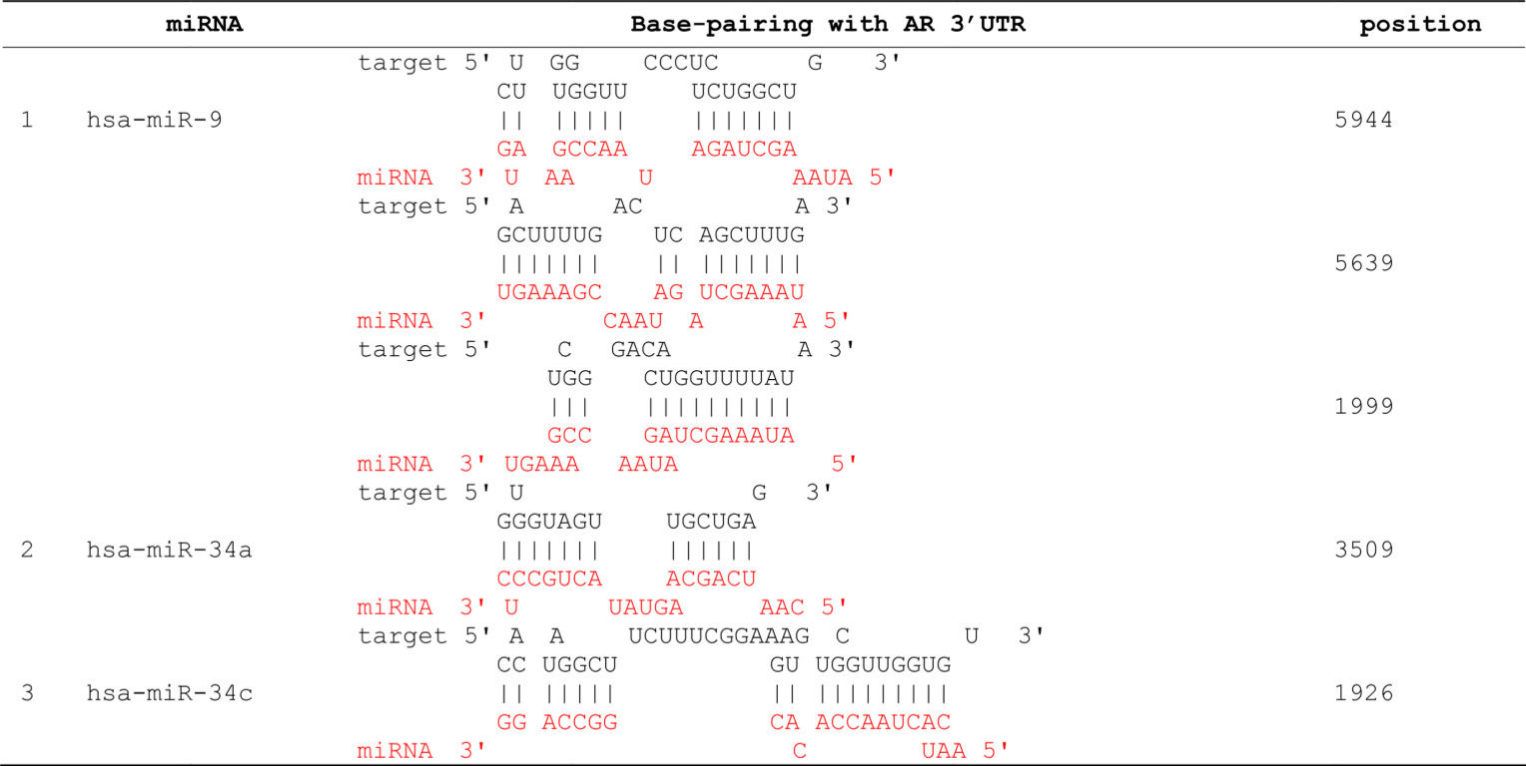
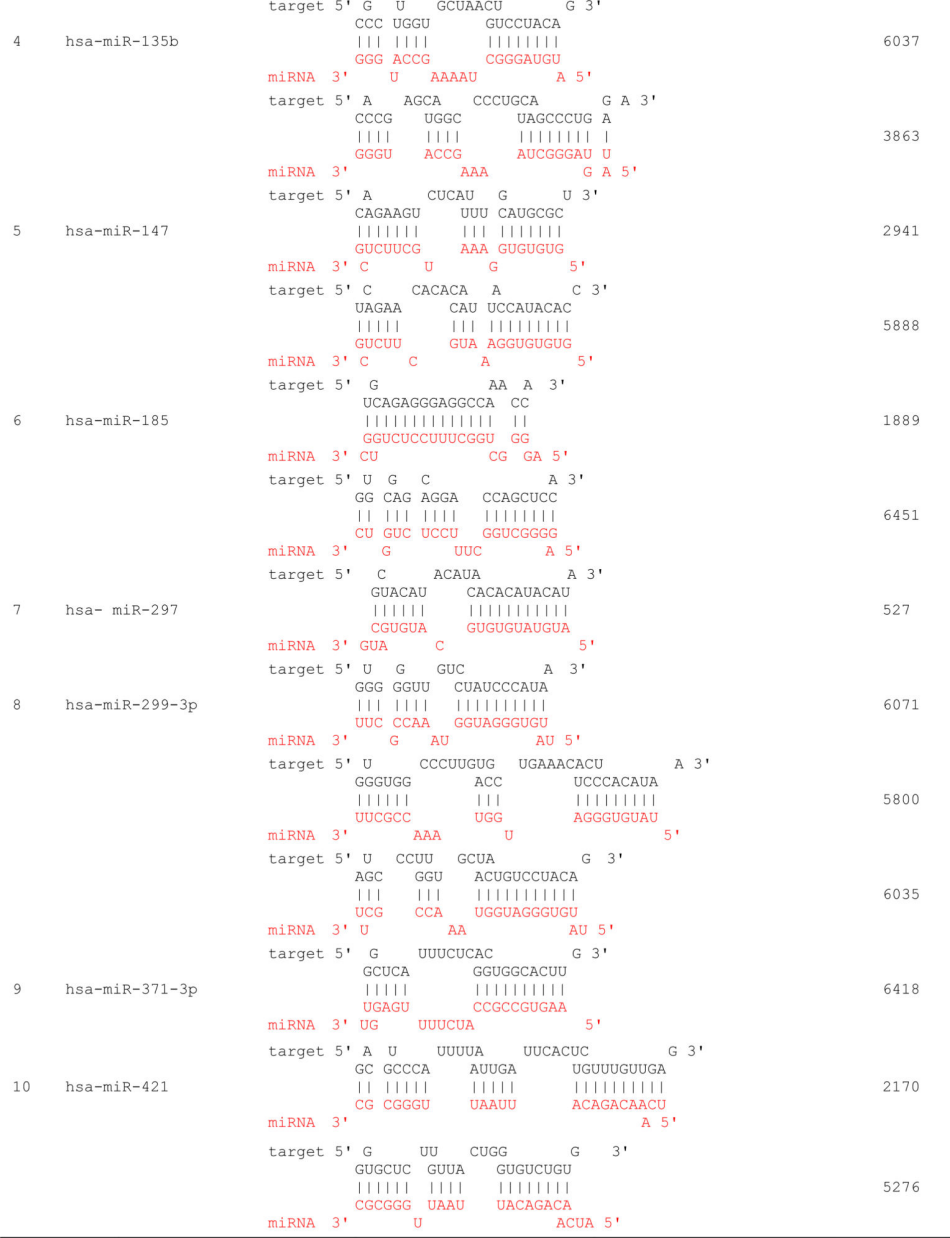
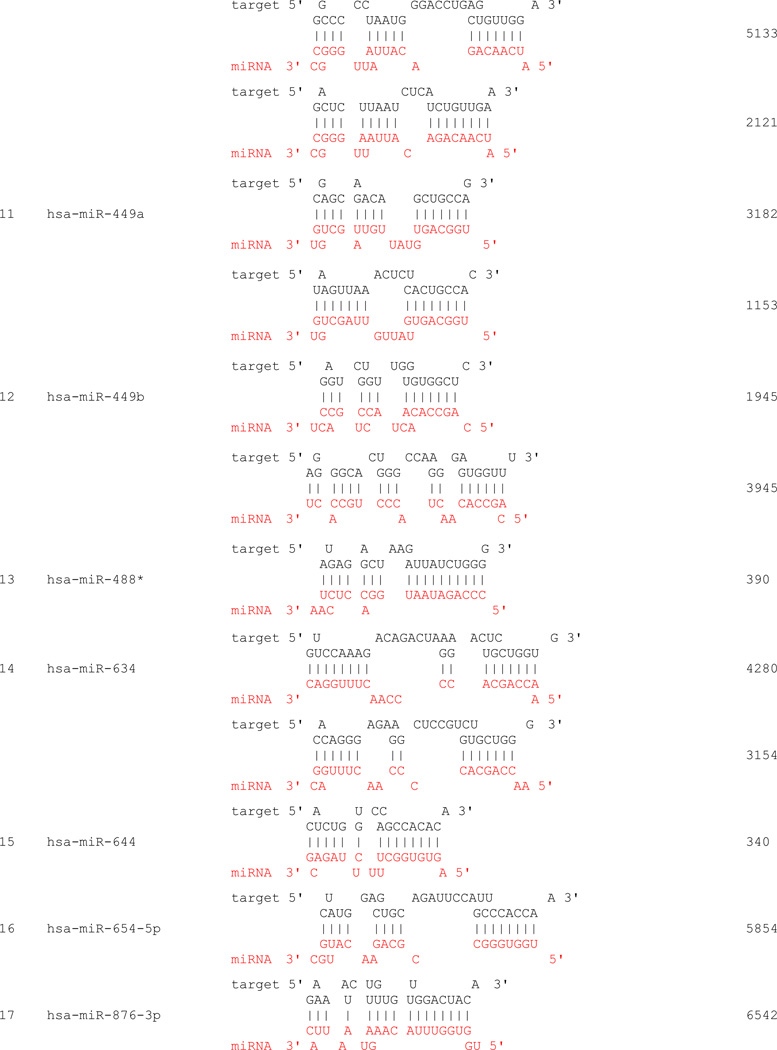

* indicates the miRNAs with increased reporter activity, though the endogenous levels of AR protein was downregulated in miRNA overexpression study of Östling P *et al.* 2011.
